# Corneal perforation with uveal prolapse: An initial presentation of orbital metastatic breast cancer

**DOI:** 10.1016/j.ajoc.2019.100551

**Published:** 2019-09-05

**Authors:** Dagmara J. Danek, Nathan W. Blessing, David T. Tse

**Affiliations:** a Lake Erie College of Osteopathic Medicine, 5000 Lakewood Ranch Blvd, Bradenton, FL, 34211, USA; b Oculoplastic and Reconstructive Surgery, Bascom Palmer Eye Institute, University of Miami, Miller School of Medicine, 900 NW 17th St, Miami, FL, 33136, USA

**Keywords:** Orbit, Metastasis, Breast cancer, Corneal perforation, Uveal prolapse, Evisceration

## Abstract

**Purpose:**

Metastasis to the orbit is a rare and typically late manifestation of a systemic malignancy. Breast cancer is the most common orbital metastatic malignancy and as the prevalence of breast cancer rises, the incidence of orbital metastasis is expected to increase concomitantly. The purpose of this report is to illustrate a unique case of orbital metastatic breast cancer with grave ophthalmic sequelae and to review the salient findings and features of orbital metastatic disease.

**Observations:**

Herein, we describe the case of a 61-year-old woman with no known history of malignancy who presented with a large compressive orbital mass that resulted in corneal perforation with uveal prolapse after initial treatment for orbital cellulitis followed by orbital pseudotumor. Anterior orbitotomy with biopsy of the mass ultimately revealed a diagnosis of metastatic breast carcinoma.

**Conclusion:**

As the incidence of breast cancer increases, ophthalmologists will play an increasingly important role in detecting both undiagnosed and recurrent breast cancer.

## 1 Introduction

Metastatic lesions represent 10% of all orbital tumors,^1^ which most commonly arise from a primary breast carcinoma.^2^ Although the orbit is a relatively rare target for malignant dissemination, orbital metastases may occur in up to 10% of patients with breast cancer.^3^ Most patients with orbital involvement are unaware as they are either asymptomatic or are being treated for metastatic symptoms elsewhere by the time visual disturbance occurs.^4^

The mean time from diagnosis of a primary breast cancer to the development of orbital metastasis is 60 months.^1^ However, in a minority of patients, visual changes from orbital metastasis may be the first sign of undiagnosed malignancy.^4^^,^^5^ As treatment for primary breast cancer has improved and the life expectancy of breast cancer patients increases, the incidence of orbital metastases is expected to rise.^5^ Ophthalmologists will play an increasingly important role in detecting both undiagnosed and recurrent metastatic breast cancer.^5^ As such, it is essential that the various signs and symptoms that may be indicative of an orbital metastatic process are recognized.

While the majority of reported cases describe intraocular breast cancer metastasis,^6^, ^7^, ^8^, ^9^, ^10^, ^11^ various presentations of extraocular metastasis have also been reported. We herein present an atypical case of extraocular orbital metastasis in a 61-year-old woman with no known history of malignancy that masqueraded and was treated initially as a case of orbital pseudotumor with devastating consequences.

## 2 Case report

A 61-year-old African American woman presented with right periorbital fullness and a perforated cornea with expulsed intraocular contents. She reported a 4 month history of progressive orbital fullness with worsening vision and eye pain over the preceding 3 weeks. She denied a history of infectious keratitis, contact lens use, ocular trauma, melanoma, lymphoma, or any other malignancy.

The patient originally presented for evaluation by an outside provider 3 months prior, after noticing blurry vision of the right eye with associated periorbital fullness and swelling for 1 month. Orbital MRI was obtained revealing an area of hyperintensity in the right orbit and an 8mm fluid collection contiguous with the superior aspect of the optic nerve sheath. The imaging findings were interpreted as being consistent with orbital cellulitis and a retrobulbar abscess and the patient was admitted for IV broad spectrum antibiotic treatment with ceftazidime, clindamycin, and vancomycin. Her visual acuity in the right eye at the time of admission was 20/200 and the globe was intact. Treatment with antibiotics was deemed ineffective and she was subsequently diagnosed with orbital inflammatory syndrome and started on systemic oral corticosteroids. A biopsy of the mass lesion was recommended but the patient was lost to follow-up.

**Fig. 1:**
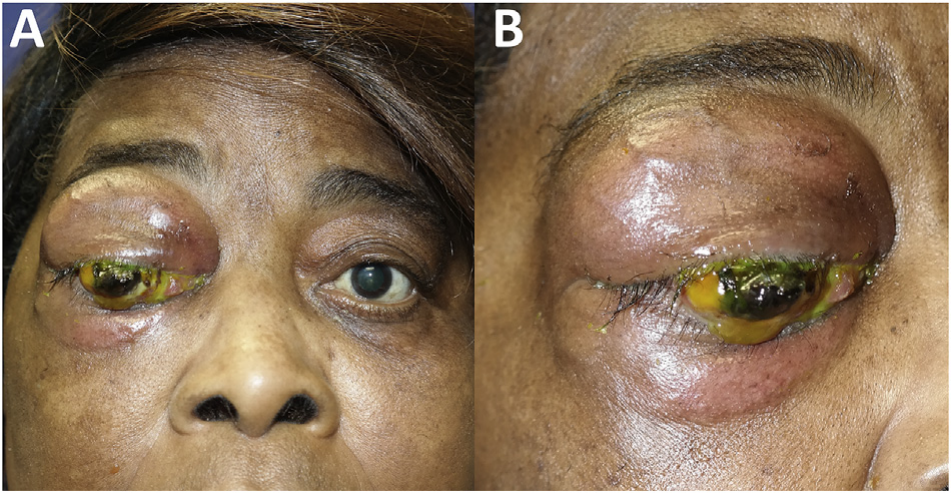
External photographs showing (A) fullness of right upper and lower eyelids, with associated proptosis and indurated erythema. A firm subcutaneous mass involving upper and lower eyelids was palpable and eye movements were restricted in all fields of gaze. (B) Conjunctiva is injected and infiltrate is seen. Uveal tissue and intraocular contents are protruding centrally through a perforated cornea.

**Fig. 2:**
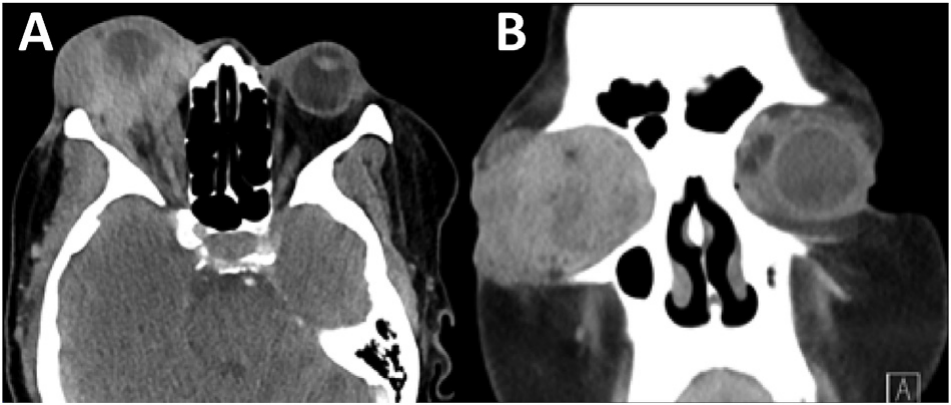
CT scan with contrast through mid-orbit. Axial (A) and coronal (B) views demonstrate a large heterogeneously enhancing mass within the right orbit, involving pre- and post-septal soft tissues. The lesion is inseparable from the lacrimal gland and encases the globe resulting in severe scleral deformation consistent with globe rupture.

Three weeks later, a total of 4 months since the onset of eye symptoms, the patient presented to Bascom Palmer Eye Institute for management of presumed panophthalmitis. The vision in the right eye was no light perception and extraocular motility was restricted in all directions of gaze. Examination revealed an inflamed and tense right orbit with palpable firmness in both the upper and lower eyelids. The cornea was perforated with expulsion of the intraocular contents (Fig. 1 A&B). Examination of the left eye was unremarkable. Imaging (Fig. 2 A & B) showed a large ill-defined orbital mass that enhanced heterogeneously with contrast. The mass encased the globe and was compressing it with severe tenting of the posterior pole. After a thorough discussion with the patient of the findings of her case she agreed to undergo evisceration of the right eye with simultaneous biopsy of the orbital mass. During surgery it was noted that the sclera was almost completely collapsed and nearly all of the intraocular contents had been expulsed. The orbital mass was dense and white without much vascularity.

**Fig. 3:**
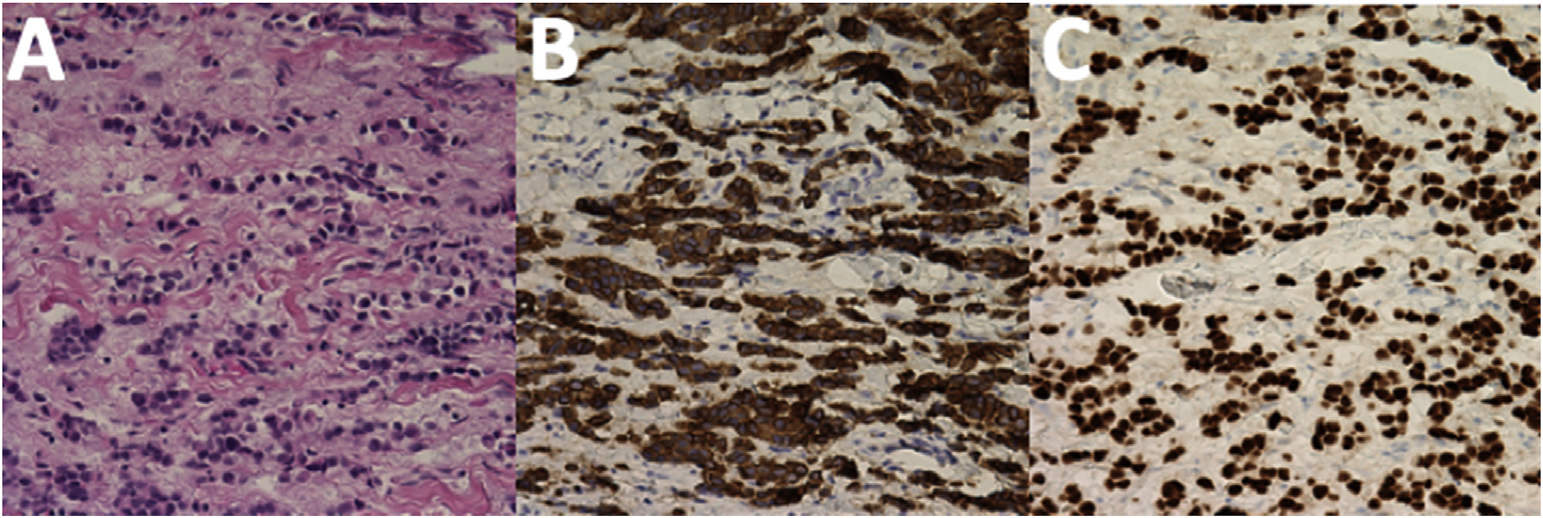
(A) H&E staining of the right orbital mass discloses fibrovascular tissue that contains atypical basophilic cells with pleomorphic nuclei and an increased nuclear to cytoplasmic ratio. The cells are variably present in a linear configuration. No organisms are identified on Gram, GMS, and AFB staining. Cytokeratin (B) and estrogen receptor (C) stains are positive. Immunohistochemical stains for Her-2 and progesterone receptors were negative. These findings are consistent with an orbital metastatic lesion from a primary breast carcinoma.

Microscopic examination of the evisceration specimen disclosed acute and chronic inflammatory cell infiltrates in the cornea, uveal tissue, vitreous, and neural retina. A cytokeratin stain for carcinoma within the intraocular contents was negative. Histopathologic examination of the orbital mass revealed a tumor comprised of atypical basophilic cells in a linear configuration with pleomorphic nuclei and an increased nucleocytoplasmic ratio (Fig. 3A). Cytokeratin and estrogen receptor stains were positive (Fig. 3B&C). These findings were consistent with an orbital metastatic lesion from a yet undetected primary breast carcinoma.

The patient suffered no intraoperative or immediate postoperative complications and the patient returned to her home country 1 week after surgery. The diagnosis was relayed to the patient and her primary care physician who arranged further evaluation with a local oncologist. Breast biopsy confirmed the diagnosis of breast carcinoma. Three months after surgery the patient was feeling well and reported an uneventful recovery period. At that time, the patient had not yet begun oncological treatment.

## 3 Discussion

Breast carcinoma accounts for up to 70% of all metastatic orbital tumors.^2^ However, because breast cancer cells preferentially spread to the lung, liver, and bones, the orbit is a rare and late metastatic site.^2^ Non-orbital involvement typically precedes orbital involvement by at least 10 months.^5^ Consequently 90% of patients already have a known diagnosis of primary breast carcinoma by the time orbital signs and symptoms manifest.^12^ For the remaining 10%, however, there is no known primary neoplasm at the time of ophthalmic evaluation.^12^ In such reported instances, a wide range of ophthalmic findings have been described (discussed in following paragraphs).^1^, ^2^, ^3^^,^^12^^,^^14^, ^15^, ^16^, ^17^, ^18^, ^19^, ^20^ However, this is the first case of orbital metastatic breast cancer to report corneal perforation with uveal prolapse as the initial presentation of malignancy.

In order to improve the detection of both undiagnosed and recurrent breast cancer, it is important for physicians to be familiar with the diverse presentations of ophthalmic metastatic disease. Intraocular involvement is more common while extraocular involvement is comparatively rare.^3^ This is primarily due to the rich vascular supply of the uveal tract wherein malignant cells are seeded hematogenously ^13^; the choroid is affected in 81% of ocular metastases.^5^ Many choroidal lesions are asymptomatic unless the macula is affected. Such patients may present with complaints of metamorphopsia and decreased vision.^4^ Signs of iris and ciliary body metastases include chronic anterior uveitis, episcleritis, and ocular hypertension due to clogging of the trabecular meshwork with malignant cells.^4^ Other intraocular structures may also be affected but generally after the uveal tract is already involved.^4^

Extraocular orbital metastases are less common, accounting for only 3%–10% of all metastases involving the eye.^14^ The extraocular muscles are most frequently involved as they also have a rich blood supply.^14^ Patients predominantly complain of pain, diplopia, and blurred vision.^14^ Reported signs typically include enophthalmos in patients with scirrhous carcinoma due to diffuse orbital fibrosis, exophthalmos in patients with particularly large masses,^15^ and restricted extraocular motility in those with muscle involvement.^14^ Globe rupture, corneal perforation, or uveal prolapse, as described in this patient, have not been reported in previously published cases of orbital metastatic breast cancer^1^, ^2^, ^3^^,^^12^^,^^14^, ^15^, ^16^, ^17^, ^18^, ^19^, ^20^, ^21^ but one patient did develop proptosis and corneal ulceration which if left untreated may have had a similar outcome to our case.^15^

Although the published prognosis of patients with orbital metastases from breast cancer is poor, with a median survival ranging from 22 to 31 months, treatment options do exist.^16^ The goals of treatment are to preserve or improve visual function, reduce pain and discomfort, and improve quality of life.^14^ Breast cancer metastases are radiosensitive and external beam radiotherapy is the most established treatment with a typical dose of 20–50 grays^14^^,^^22^ Radiotherapy is a widely available treatment modality and a study by Wiegel et al. found that this treatment stabilized or restored vision in up to 86% of patients.^4^ Reported complications include radiation-induced conjunctivitis, cataracts, exposure keratopathy, retinopathy, optic neuropathy, and iris neovascularization.^14^^,^^22^^,^^23^

Other palliative treatment options include systemic chemotherapy, plaque brachytherapy, hormonal therapy, and tumor debulking surgery.^14^^,^^21^ Enucleation is reserved for intractable ocular pain from complications of end-stage disease including chronic glaucoma, phthisis bulbi, persistent tumor growth, or globe rupture.^14^^,^^23^ The choice of therapy depends on the location and extent of orbital metastases, as well as the patient's medical condition and life expectancy.^4^

As the life expectancy of breast cancer patients continues to increase, the incidence of both intraocular and extraocular metastases is also expected to rise. It is important for physicians to include metastatic disease in the differential diagnosis when suggestive ocular signs and symptoms appear. Additionally, although the median interval between initial breast cancer diagnosis and orbital metastases is 5 years, it should be noted that ophthalmic findings precede detection of a primary neoplasm in approximately 10% of affected patients.^4^^,^^12^ Orbital metastasis should be on the differential diagnosis of any patient with an orbital mass.

Demographic patient features can help to guide clinical examination in searching for a primary malignancy even in the absence of confirmatory histopathologic information. Earlier diagnosis, even that of advanced metastatic disease, results in fewer complications, more favorable treatment outcomes, and an improved quality of life.^4^

## Patient consent

Written consent to publish case details and photographs was obtained from the patient.

## Funding

This work was supported in part by: NIH Center Core Grant P30EY014801; Research to Prevent Blindness Unrestricted Grant, Inc, New York, New York; and the Dr. Nasser Ibrahim Al-Rashid Orbital Vision Research Fund. The sponsor or funding organization had no role in the design or conduct of this research.

## Conflicts of interest

No conflicting relationship exists for any author: DD, NB, DT.

## Authorship

All authors attest that they meet the current ICMJE criteria for Authorship.
